# Assessment of the Effective Management of Patients With Severe Primary Hypercholesterolemia Under Care in Family Medicine Clinics at King Faisal Specialist Hospital and Research Centre, Riyadh, Saudi Arabia

**DOI:** 10.7759/cureus.30701

**Published:** 2022-10-26

**Authors:** Ghada Hussein, Muna S Albashari, Hadeel M Alarfaj, Abdelrafour Houdane, Zainab Wagley, Atheer A Alsaleh, Yaser A Alendijani

**Affiliations:** 1 Family Medicine, King Faisal Specialist Hospital and Research Centre, Riyadh, SAU; 2 Family Medicine, Alfaisal University College of Medicine, Riyadh, SAU

**Keywords:** ldl-c, lipid lowering, family medicine, atherosclerotic cardiovascular disease, hypercholesterolemia

## Abstract

Background

Atherosclerotic cardiovascular disease (ASCVD) is the primary cause of death in Saudi Arabia. Hypercholesterolemia is a prevalent risk factor that can lead to ASCVD. The American College of Cardiology/American Heart Association (ACC/AHA) guidelines have provided recommendations for managing severe primary hypercholesterolemia, defined as medically well adults 21-75 years of age with low-density lipoprotein cholesterol (LDL-C) ≥190 mg/dL (≥4.9 mmol/L). Underutilization of the guideline recommendations has led to concern and the need for further review. This study aims to review the management of severe primary hypercholesterolemia in the Family Medicine and Polyclinics at King Faisal Specialist Hospital and Research Centre (KFSH&RC) in Riyadh, Saudi Arabia.

Methodology

In this retrospective cohort study, data were obtained from electronic medical records of patients aged 21-75 years who received care in the Family Medicine and Polyclinics at KFSH&RC in Riyadh with LDL-C ≥190 (≥4.9 mmol/L). The data collected included demographics, body mass index (BMI), LDL-C blood level, and lipid-lowering medications prescribed. We measured the prevalence of hypercholesterolemia, reviewed if appropriate statin therapy was prescribed as per the ACC/AHA guidelines, and determined if treated patients with severe primary hypercholesterolemia achieved LDL-C ≤100 mg/dL (≤2.6 mmol/L) from January 1, 2015, until June 30, 2020.

Results

The prevalence of hypercholesterolemia was 7.4%. The sample size studied included 195 patients. The majority of patients were aged 40-59 years and were either overweight or obese. Treatment with a moderate-intensity statin was observed in 46.4% of patients, and 45.4% of patients were not prescribed a statin. The LDL-C ≤100 mg/dL (≤2.6 mmol/L) was not achieved in 88.3% of patients.

Conclusions

Despite guidelines, the majority of patients with severe primary hypercholesterolemia are inadequately managed. High-risk patients need to be diagnosed appropriately so that they receive proper treatment to prevent ASCVD. We encourage adherence to established guidelines in the management of severe primary hypercholesterolemia to prevent premature ASCVD.

## Introduction

Atherosclerotic cardiovascular disease (ASCVD) is a major health concern worldwide. Studies have shown that ASCVD likely leads to 22-42% of all deaths in Saudi Arabia [1]. High blood cholesterol levels are a major risk factor that, if left untreated, contributes to the development of ASCVD. Hypercholesterolemia is highly prevalent in the Saudi Arabian population, with estimates as high as 50% in some areas [2]. Appropriately managing patients with high blood cholesterol levels can prevent morbidity and mortality. Because there are no local clinical practice guidelines in Saudi Arabia on the management of dyslipidemia, most healthcare providers refer to international guidelines such as the American College of Cardiology/American Heart Association (ACC/AHA) guidelines [3]. Management of patients with high blood cholesterol levels varies even though there are research-based guidelines addressing this disease.

The ACC/AHA Task Force has provided clinical guidelines on the management of blood cholesterol to address this worldwide concern to reduce the risk of ASCVD [4]. Although the guidelines focus on American medical practice, they are relevant to patients throughout the world [5]. Patients with severe primary hypercholesterolemia, defined as low-density lipoprotein cholesterol (LDL-C) levels greater than or equal to 190 mg/dL (≥4.9 mmol/L), have a fivefold increased lifetime risk of heart disease and develop heart disease 10-20 years earlier [6]. The 2013 ACC/AHA guidelines recommend high-intensity statin therapy for patients aged 21-75 with severe primary hypercholesterolemia. ASCVD risk scoring is not needed for patients with severe primary hypercholesterolemia due to the strong data demonstrating the reduced risk of ASCVD with treatment [4]. The 2013 ACC/AHA guidelines did not recommend a specific LDL-C goal; however, patients with severe primary hypercholesterolemia have a high lifetime risk for ASCVD for which intensive management and substantial reduction in LDL-C is warranted [4]. The ACC/AHA guidelines were updated in 2018, and a target goal of LDL-C ≤100 mg/dL (≤2.6 mmol/L) was recommended in the management of patients with severe primary hypercholesterolemia.

Studies in Saudi Arabia reported that more than a million Saudis were hypercholesterolemic, and 0.7 million of them were unaware of their condition [3]. Compared to Western countries, the Middle Eastern population has earlier-onset ASCVD [3]; therefore, cumulative benefit with treatment and any lowering of LDL-C is beneficial [7]. Due to the high prevalence of hypercholesterolemia that is inadequately treated, there is an urgent need to raise awareness among primary care providers of the importance of implementing the ACC/AHA guidelines in patients with severe primary hypercholesterolemia to reduce ASCVD risk.

Our objectives in this study are to measure the prevalence of patients 21-75 years of age with hypercholesterolemia, evaluate the appropriateness of prescribed statin therapy as per the ACC/AHA guidelines in patients with severe primary hypercholesterolemia, and determine if patients treated for severe primary hypercholesterolemia achieved target LDL-C levels.

## Materials and methods

Electronic health records of medically well patients aged 21-75 years who received care at King Faisal Specialist Hospital and Research Centre (KFSH&RC) Family Medicine and Polyclinics were reviewed from the Integrated Clinical Information System (ICIS) database retrospectively from January 1, 2015, through June 30, 2020. Patients with secondary causes such as nephrotic syndrome, cholestasis, or hypothyroidism were excluded. Patients who received Roaccutane, pregnant or postpartum women, those who had abnormal liver function tests, or patients already on statin therapy were excluded. Data collected included demographics such as age, gender, nationality, body mass index (BMI) (i.e., normal: BMI ≤24.9 kg/m^2^, overweight: BMI 25-29.9 kg/m^2^, obese: BMI ≥30 kg/m^2^), current tobacco use as per chart review (i.e., non-smoker includes ex-smokers), LDL-C level at the time of diagnosis, LDL-C at the last visit or end of the study, statin intensity prescribed after diagnosis, dosage changes for statin, and other lipid-lowering medications prescribed (i.e., ezetimibe, fenofibrate, gemfibrozil, or niacin).

According to the ACC/AHA guidelines, statin intensity was defined as high (i.e., atorvastatin 40 or 80 mg or rosuvastatin 20 or 40 mg), moderate (i.e., atorvastatin 10 or 20 mg or rosuvastatin 5 or 10 mg or simvastatin 20 or 40 mg) and low (i.e., simvastatin 10 mg).

All data were analyzed using the SPSS software package version 26.0 (IBM Corp., Armonk, NY, USA). Descriptive statistics for the categorical variables were summarized as frequencies and percentages. Additionally, inferential statistics were performed on categorical variables using the Chi-square test and Fisher’s exact test to compare the differences among LDL-C changes as per the ACC/AHA guidelines. Continuous variables including LDL-C and age were categorized and compared as categorical variables. The significance level was set at 0.05 and 95% confidence interval (CI).

The research project was conducted in accordance with the ethical principles contained in the Declaration of Helsinki (2000), the WHO Operational Guidelines for Ethical Committees that review Biomedical research (2000), the International Ethical Guidelines for biomedical research involving human subjects (2002), and the policies of the Research Advisory Committee (RAC) at KFSH&RC, as well as the laws of the Kingdom of Saudi Arabia. The RAC at KFSH&RC reviewed and approved this research (RAC #2221016).

## Results

Of the 14,940 patients seen in the Family Medicine and Polyclinics at KFSH&RC from 2015 through 2020, 1,113 patients had LDL-C ≥4.9 mmol/L without applying the study’s eligibility criteria, resulting in a prevalence of 7.4%. After all of the files were reviewed, 195 of the 1,113 patients fit the study’s criteria and were analyzed.

Data from 195 patients who fit the criteria of the study were collected. There was an almost equal distribution of non-Saudi (49.5%) and Saudi patients (50.5%), as shown in Table 1. We found that 188 (95.9%) patients lived in Riyadh. Most patients were either overweight (42.7%) or obese (35.9%). Mainly, patients were non-smokers (83.4%). The highest prevalence was in the category from 40 through 59 years of age (Table 1). Older adults, from 60 through 75 years of age (15.3%), comprised the least studied age group.

**Table 1:** Demographic characteristics of patients included in the study. BMI: body mass index

Variable	Characteristics	Frequency (N = 196)	Percentage
Nationality	Non-Saudi	96	49.5
	Saudi	99	50.5
Gender	Female	107	55.1
	Male	88	44.9
BMI	Normal	38	21.4
	Overweight	78	42.7
	Obese	62	35.9
Smoking status	Smoker	31	16.6
	Non-smoker	151	83.4
Age (years)	<40	50	25.5
	40–59	59	30.1
	50–59	57	29.1
	60–75	30	15.3

**Figure 1:**
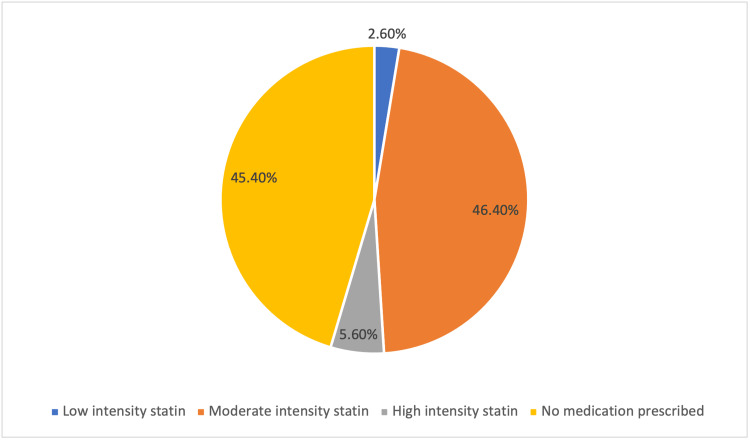
Percentage of patients prescribed lipid-lowering medication.

The majority of patients were either prescribed moderate-intensity statins (46.4%) or not prescribed any lipid-lowering medication (45.4%), as shown in Figure 1. A high-intensity statin was prescribed at 5.6%. The use of other lipid-lowering medications such as ezetimibe and fenofibrate was minimal: 11 (5.6%) patients and three (1.5%) patients, respectively. The statin dosage was changed in 52 (26.5%) patients; of those who had their dosage changed, 43 (21.9%) patients had their dosage increased. In our sample, six (3.1%) of the patients experienced side effects from the statin, so the dosage was changed.

Follow-up clinic appointments were observed for the majority of patients (170, or 89.9%) after the initial visit of high LDL-C with repeat testing. We analyzed the data to determine if the goal less than or equal to LDL-C 2.6 mmol/L was achieved by the end of the study (Table 2). Throughout all age groups, the target LDL-C level was not achieved. Although goal LDL-C was not achieved (88.3%), 138 (70.4%) patients were noted to have a decrease in end-of-study LDL-C from the first test result. No statistical differences were observed in achieving LDL-C ≤2.6 mmol/L and age (p = 0.380), gender (p = 0.455), nationality (p = 0.133), BMI (p = 0.095), or smoking (p = 0.999). There was a statistically significant relationship between gender and LDL-C level change (p = 0.031). When statin dosage was changed, there was a statistically significant difference in LDL-C level change (p = 0.016).

**Table 2:** Analysis of variables in relation to the achievement of LDL-C ≤2.6 mmol/L. BMI: body mass index; LDL-C: low-density lipoprotein cholesterol

Variable	Was the last LDL-C level ≤2.6 mmol/L?		P-value
	Yes	No	
Age (years)			0.380
<40	3 (13%)	47 (27.2%)	
40–49	9 (39.1%)	50 (28.9%)	
50–59	6 (26.1%)	51 (29.5%)	
≥60	5 (21.7%)	25 (14.5%)	
Gender			0.455
Male	12 (52.2%)	76 (43.9%)	
Female	11 (47.8%)	97 (56.1%)	
Nationality			0.133
Saudi	15 (65.2%)	84 (48.6%)	
Non-Saudi	8 (34.8%)	89 (51.4%)	
BMI			0.095
Normal weight	3 (13%)	38 (22.5%)	
Overweight	15 (65.2%)	67 (39.6%)	
Obese	5 (21.7%)	64 (37.9%)	
Smoking			0.999
Smoker	3 (13.6%)	29 (17%)	
Non-smoker	19 (86.4%)	142 (83%)	
Prescribed statin intensity			0.711
High intensity	1 (4.3%)	10 (5.8%)	
Moderate intensity	12 (52.2%)	79 (45.7%)	
Low intensity	1 (4.3%)	4 (2.3%)	
Not prescribed	9 (39.1%)	80 (46.2%)	
Statin dose changed			0.339
Yes	9 (39.1%)	43 (29.3%)	
No	14 (60.9%)	104 (70.7%)	

## Discussion

The prevalence of hypercholesterolemia in our study was 7.4%, not excluding patients with medical conditions. The prevalence varies in different populations with previous large sample studies reporting the prevalence to range from 8% to over 50% [8]. Our study sample was small; the objective was to determine the prevalence of hypercholesterolemia, which is defined by the 2013 ACC/AHA guidelines as medically well patients aged ≥21 years old with LDL-C ≥4.9 mmol/L. We excluded patients who were already taking lipid-lowering medications and those with medical conditions that could lead to an underestimated prevalence. To our knowledge, this was the first study to review the management of severe primary hypercholesterolemia in Saudi Arabia.

Although there was an equal number of patients in the study who were Saudi and non-Saudi and of the opposite gender, no statistical significance was observed. Obese and overweight patients comprised the majority of the patients studied, this is a risk-enhancing factor that can accelerate ASCVD. We analyzed the data of Saudi and non-Saudi patients because KFSH&RC has many expatriate employees, but there were no significant differences or associations between LDL-C ≥4.9 mmol/L and nationality, gender, BMI, or smoking status. Nearly 25% of patients in the cohort were under 40 years of age, as the progression of ASCVD occurs in middle age or later years, more aggressive management of hypercholesterolemia is warranted [5]. ASCVD is multifactorial; however, we did not analyze the association of physical activity, dietary intake, family history of premature ASCVD, or genetic forms of hypercholesterolemia in our study.

As per the ACC/AHA guidelines, high-intensity statin therapy is recommended for the treatment of severe primary hypercholesterolemia with LDL-C ≥4.9 mmol/L [4]. Greater ASCVD risk reduction is seen in adherent patients prescribed high-intensity versus moderate-intensity statin therapy [4]. Unfortunately, high-intensity statins were only prescribed to 5.6% of patients studied; furthermore, the only option at KFSH&RC for high-intensity statin is atorvastatin. The management of severe hypercholesterolemia varied, despite guidelines; 46.4% were prescribed moderate-intensity statins, and 45.4% were not prescribed any lipid-lowering medication. Similarly, a large retrospective study in the United Kingdom of 205,040 patients reported moderate-intensity statin use at 69.3% as monotherapy for hypercholesterolemia [9]. Non-statin medications, ezetimibe and fenofibrate, were prescribed in 7.7% of patients, although they are not recommended as monotherapy for the treatment of severe primary hypercholesterolemia. Furthermore, the ACC/AHA guidelines recommend non-statin medications as add-on therapy to statins to lower LDL-C under certain circumstances [4].

The National Institute for Health and Care Excellence (NICE) is another evidence-based guideline utilized by physicians at KFSH&RC. The NICE guidelines recommend using the QRISK2 risk assessment tool to assess cardiovascular disease (CVD) risk for primary prevention of CVD in people up to 84 years old [10]. European guidelines recommend the use of the SCORE system in clinical practice to estimate cardiovascular risk and make treatment recommendations based on the risk [11]. The lack of similar recommendations regarding the treatment of severe primary hypercholesterolemia among different guidelines has led to the variation in management used in clinical practice and is a gap in care that needs to be addressed further.

The 2018 ACC/AHA guidelines recommend an LDL-C target level of less than or equal to 2.6 mmol/L after statin treatment. Statin therapy was increased in 21.9% of patients, while 3.1% of patients experienced side effects that warranted a decrease in statin dosage. Unfortunately, the target goal was not achieved in the majority of patients in the study; nonetheless, there was a decrease in LDL-C levels in 70.4% of the patients. Limitations to the achievement of the target goal can be due to the differences in the duration of follow-up testing after the first result, a lack of follow-up testing, and patient adherence.

Combination therapy was underutilized in our study sample, despite being available and an option in the treatment of patients not achieving their LDL-C target goal. Proprotein convertase subtilisin/Kexin type 9 (PCSK9) was not prescribed, despite the fact that this class of medication has shown to be beneficial in selected maximally treated patients with persistently elevated LDL-C [5]. The lack of PCSK9 use could be attributed to a lack of availability and restricted prescribing to specialists at the time of the study.

## Conclusions

Hypercholesterolemia is a major risk factor for ASCVD that needs to be diagnosed and treated aggressively through lifestyle changes and/or medications. Primary severe hypercholesterolemia patients have a five times higher risk for ASCVD and are poorly managed. Worldwide, this is a growing medical concern, and research suggests that target LDL-C levels are not achieved, especially throughout the Middle East.

Risk assessment guidelines are available, so it is imperative that medical providers are aware of them and follow the recommendations. Screening, monitoring, and medication therapy adjustments along with shared decision-making will improve the management of hypercholesterolemia. More awareness, standardization of guidelines, and education are needed to address the gaps in the management of severe primary hypercholesterolemia to prevent premature ASCVD.
